# The effect of agomelatine and melatonin on sleep-related eating: a case report

**DOI:** 10.1186/s13256-017-1438-5

**Published:** 2017-09-28

**Authors:** Angela Alexandra Zapp, Eva Caroline Fischer, Michael Deuschle

**Affiliations:** 1 Central Institute of Mental Health, Department of Psychiatry and Psychotherapy, University of Heidelberg, Faculty of Medicine Mannheim, J5, 68159 Mannheim, Germany; 2 University Hospital Würzburg, University of Würzburg, Würzburg, Germany

**Keywords:** Sleep-related eating, Agomelatine, Melatonin, Weight loss, Parasomnia

## Abstract

**Background:**

Sleep-related eating may occur in the context of mental illness, sleep disorders, or psychopharmacological treatment. Frequently, sleep-related eating leads to severe weight gain and, so far, there are no treatment options for the condition.

**Case presentation:**

We report the case of a 54-year-old white woman with depression, panic disorder, and sleep apnea under treatment with various antidepressants who developed severe sleep-related eating. Her sleep-related eating completely vanished after addition of agomelatine, it reoccurred after cessation of agomelatine, and vanished again after her re-exposure to another melatonergic drug, extended melatonin.

**Conclusions:**

This case suggests that melatonergic drugs lead to relief from sleep-related eating, even when the condition occurs in the context of physical and mental disorders as well as psychopharmacological treatment.

## Background

In humans, eating and sleeping are organized in a circadian manner. In the case of sleep-related eating (SRE), this circadian regulation is disturbed and uncontrollable eating occurs during nighttime sleep. Episodes of SRE mainly take place upon transition from sleep to wakefulness. Usually, there is reduced consciousness and in the morning there might be no recall of the nighttime eating episodes [1]. The disorder mainly occurs in non-rapid eye movement (NREM) sleep [2] and SRE is considered a parasomnia disorder, frequently associated with sleepwalking [3]. Patients often have undesirable weight gain due to a preference for high-calorie food during SRE and as a result have health consequences.

The American Academy of Sleep Medicine operationalized SRE disorder (SRED) as:

1. Recurrent episodes of dysfunctional eating that occur after an arousal during the main sleep period.
2. The presence of
  - the consumption of peculiar forms or combinations of food or inedible or toxic substances or
  - sleep-related injurious or potentially injurious behaviors performed while in pursuit of food or while cooking food or
  - adverse health consequences from recurrent nocturnal eating.
3. The partial or complete loss of conscious awareness during the eating episode with subsequent impaired recall.
4. The disturbance is not better explained by another sleep, mental, or medical disorder, medication, or substance use [4].

A SRED is more common in patients with sleep disorders like sleepwalking, periodic limb movement, restless legs syndrome, and sleep apnea or mental conditions like posttraumatic stress disorder or major depressive disorder. Phase delays in melatonin secretion suggest that SRE should be considered a circadian condition. Moreover, there are cases of SRE under treatment with various psychopharmacological agents (for review see [1]).

Usually SRED is treated with selective serotonin reuptake inhibitors at mean dosages. Topiramate at 100 to 300 mg/day and clonazepam at 0.5 to 2.0 mg/day can be valid alternative options [5]. Melatonergic drugs were tried on night eating syndrome but to our current knowledge they have not been tried on SRED [6]. This study was to determine if there is a benefit of melatonergic drugs on SRED.

## Case presentation

In June 2016 a 54-year-old white woman was admitted for treatment of her chronic major depressive episode in the course of a major depressive disorder as well as panic disorder. She complained of severely depressed mood, loss of pleasure and interest, loss of drive and energy, disturbed sleep, and increased weight. The current depressive syndrome began approximately 5 years before admission. Her first episode occurred 36 years ago at the age of 18. Moreover, she had spontaneous and situationally induced panic attacks with severe avoiding behavior that did not allow her to leave the house by herself for at least 2 years. She lives with her husband (married for 22 years) in a house and has two adult children.

She has hypertension, which is treated with 5 mg ramipril, and adiposity. Between the age of 10 and 16 she sleepwalked once a month. No other physical illness is known.

Ten years ago a therapy with doxepin, venlafaxine, and lithium was started. Episodes of dysfunctional eating at nighttime made their first appearance in the course of this treatment. Observed by her husband approximately once or twice a week she had nightly eating and SRE, mostly without any recall in the morning. During these episodes, she ate large amounts of unusual foods (glass jars of marmalade, several bars of chocolate, and so on), brought food into her bed, and gained approximately 20 kg in the last years. SRE started with a frequency of approximately once a week and continued after the cessation of drug treatment. From 2015 to 2016 she took doxepin once or twice a week when she felt sleepless. Besides this she was on no other medication. During these nights with intake of doxepin she noticed SRE events. She became aware of the nightly eating through food wrappings in her bed and her husband confirmed the consumption through observance. Her body mass index (BMI) was 48.8 kg/m^2^ at admission to our hospital. She had normal blood values except for cholesterol (217 mg/dl), C-reactive protein (CRP; 17.4 mg/l; permanently until discharge), gamma-glutamyltransferase (GGT; 44 U/l), and blood glucose (123 mg/dl). Urine analysis was within normal limits. Her blood pressure was 140/90 mmHg under medication with ramipril 5 mg. An electroencephalogram (EEG), electrocardiogram (ECG), and thyroid scintigraphy showed no pathological result. A magnetic resonance imaging (MRI) was refused on the basis of her panic disorder and accompanying anxieties.

A head, eye, ear, nose, and throat physical examination was within normal limits. A chest examination was clear to auscultation bilaterally. An examination of her heart was notable for a normal S1, S2, and it was without rubs, murmurs, or gallops. Her pulse was 84 beats/minute. Her abdomen was soft, obese, with no organomegaly, and normoactive bowel sounds in all quadrants. A neurologic examination revealed that all her cranial nerves were grossly intact. Her strength was 5/5 throughout with 2+ reflexes. Her sensation to fine touch was intact throughout.

**Fig. 1:**
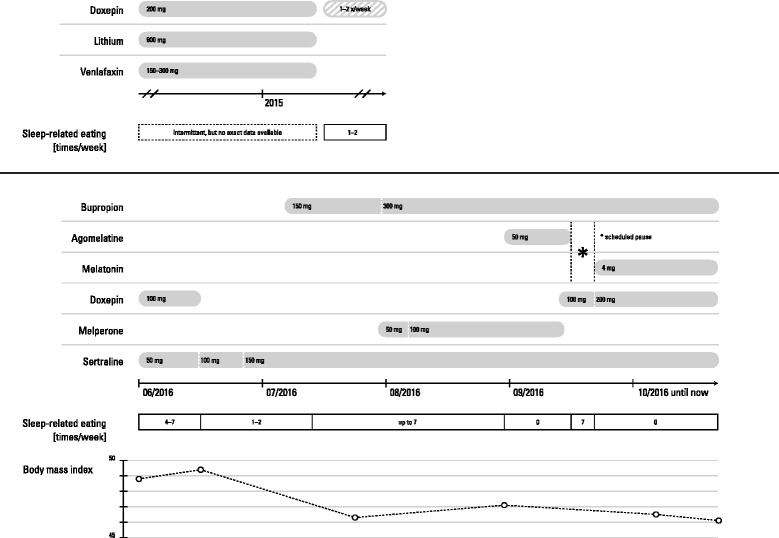
Chronological correlation of drug therapy, weight loss, and sleep-related eating. Graphic created by Dr Johannes Taeger

In our hospital, SRE continued under antidepressant treatment with sertraline plus doxepin. Under therapy with 150 mg sertraline, 50 to 100 mg melperone, and 150 to 300 mg bupropion the frequency of nighttime eating increased to at least once a night (Fig. 1)

With regard to sleep disorders, in the presence of severe adiposity, we found evidence for a moderate sleep apnea syndrome with an apnea–hypopnea index of 6.9 per hour in polygraphy. She complained about an irresistible urge to move her legs almost exclusively during nighttime, with temporary relief from this urge during movements, but had no sensations of pain or other unpleasant sensations and, therefore, did not completely fulfil the criteria for restless legs syndrome.

In order to treat SRE we added 50 mg agomelatine, which is in accordance with reports of treatment of a similar disorder, nighttime eating [6]. During the following 14 nights, SRE completely vanished and reoccurred after stopping agomelatine for a week. Then we added 4 mg melatonin extended release and again, immediately, SRE vanished and she lost 3 kg of weight in the following 3 weeks. Her BMI fell to 46.5 kg/m^2^ and she lost 6.1 kg in total at discharge from our hospital. A follow-up 2 months after discharge showed a further reduction of her BMI to 45.7 kg/m^2^. At the second follow-up 10 months after discharge we found that she remained well until 5 months after discharge and then developed a further episode of major depression mainly attended by avoidance behavior. She refused complementary individual and group psychotherapy as well as her support group. A voracious appetite emerged during the day and she consumed a huge amount of fast food through which she gained weight up to a BMI of 49.4 kg/m^2^. Nightly eating occurred up to once a week from that time on but never again reached a level of seven times a week as in August 2016.

## Discussion

Our patient had a clear syndrome of SRE. The syndrome started before the onset of the current depressive episode and its treatment. Moreover, the frequency of SRE was associated with antidepressant treatment, especially doxepin, sertraline, and bupropion. In addition, she had a mild sleep apnea syndrome and fulfilled three out of four criteria of restless legs syndrome. Therefore, the condition might have, at least partly, been due to a medical condition. In fact, nighttime eating disorders are frequently associated with mental disorders, for example affective disorders, as well as sleep disorders [7] or treatment with antidepressants. So far, there is evidence from a case series that agomelatine may ameliorate night eating [6]. Since agomelatine affects the melatonergic as well as the serotonergic system, the exact mode of action remained unclear. Our case showed complete recovery from SRE with agomelatine as well as melatonin. These findings suggest (1) that the melatonergic properties of agomelatine may mediate the SRE-suppressing effect, (2) that melatonin is a potential alternative to agomelatine treatment of SRE, and (3) that even in the presence of other causes, like affective or sleep disorders (here, depression, panic disorder, sleep apnea), and psychopharmacological treatment, a treatment using a circadian mechanism is effective. To our current knowledge melatonergic drugs have not been tried on SRED before. Clearly, a placebo-controlled study with melatonin in patients with SRE is warranted.

## Conclusions

This case suggests that melatonergic drugs lead to relief from SRE, even when the condition occurs in the context of physical and mental disorders as well as psychopharmacological treatment.
